# Comparison of the neutralization sensitivity of South African and Indian HIV-1 subtype C viruses to South African plasma antibodies

**DOI:** 10.1186/1742-4690-9-S2-P66

**Published:** 2012-09-13

**Authors:** T Hermanus, N Mkhize, P Moore, M Nonyane, J Battacharya, SA Karim, L Morris

**Affiliations:** 1 National Institute for Communicable Diseases, Johannesburg, South Africa; 2 National AIDS Research Institute, Pune, India; 3 Center for AIDS Program of Research in South Africa, Durban, South Africa

## Background

HIV-1 subtype C is the predominant subtype in India and South Africa (SA) responsible for explosive epidemics. An understanding of the neutralization sensitivity of viruses from these regions will help to define shared epitopes in the envelope protein, which may be important for inclusion in an effective subtype C-specific vaccine.

## Methods

Plasma from 44 chronically HIV-1 subtype C infected SA individuals in the CAPRISA 002 Acute Infection cohort were used to assess the neutralization sensitivity of 10 SA and 9 Indian Tier 2 viruses in a TZM-bl assay. Intra-subtype neutralization activity between SA and Indian viruses was determined. The envelope sequence of the Indian panel was analyzed in order to identify epitopes known to be targeted by the neutralizing antibodies in a subset of the CAPRISA plasmas.

## Results

South African and Indian viruses were generally sensitive to the most potent plasmas. However, SA viruses showed overall greater sensitivity compared to Indian viruses; neutralization of 50% of the SA virus panel was achieved by 15/44 (34%) of the plasmas compared to only 6/44 (14%) against the Indian panel. Furthermore, neutralization titers against SA viruses were higher, with 9/44 (21%) of plasmas neutralizing at >1:200 compared to 5/44 (11%) against the Indian panel. An analysis of the gp160 sequences suggested that genetic differences in key neutralization epitopes between SA and Indian viruses were in part responsible for differences in sensitivity.

## Conclusion

These data suggest the presence of common neutralization epitopes between SA and Indian viruses although there is also evidence for regional neutralization determinants within subtype C. The finding that SA viruses were generally neutralized at higher titers, suggests that the exposure of common epitopes on envelope may vary between SA and Indian viruses. Overall, these data support the development of a subtype C-specific vaccine that can be used in both SA and India.

